# Plutonium dioxide particle imaging using a high-resolution alpha imager for radiation protection

**DOI:** 10.1038/s41598-021-84515-z

**Published:** 2021-03-15

**Authors:** Yuki Morishita, Shunsuke Kurosawa, Akihiro Yamaji, Masateru Hayashi, Makoto Sasano, Taisuke Makita, Tetsushi Azuma

**Affiliations:** 1 Collaborative Laboratories for Advanced Decommissioning Science (CLADS), Japan Atomic Energy Agency, 790-1 Motooka Ohtsuka, Tomioka Town, Futaba-gun, Fukushima, 979-1151 Japan; 2 New Industry Creation Hatchery Center (NICHe), Tohoku University, 6-6-10 AobaAoba-ku, AramakiSendai, Miyagi 980-8579 Japan; 3 Institute for Materials Research (IMR), Tohoku University, 2-1-1 Katahira, Aoba-ku, Sendai, Miyagi 980-8577 Japan; 4 Advanced Technology R&D Center, Mitsubishi Electric Corporation, 8-1-1, Tsukaguchi-honmachi, Amagasaki City, Hyogo, 661-8661 Japan

**Keywords:** Nuclear physics, Nuclear fuel, Environmental monitoring, Imaging, Imaging and sensing

## Abstract

The internal exposure of workers who inhale plutonium dioxide particles in nuclear facilities is a crucial matter for human protection from radiation. To determine the activity median aerodynamic diameter values at the working sites of nuclear facilities in real time, we developed a high-resolution alpha imager using a ZnS(Ag) scintillator sheet, an optical microscope, and an electron-multiplying charge-coupled device camera. Then, we designed and applied a setup to measure a plutonium dioxide particle and identify the locations of the individual alpha particles in real time. Employing a Gaussian fitting, we evaluated the average spatial resolution of the multiple alpha particles was evaluated to be 16.2 ± 2.2 μmFWHM with a zoom range of 5 ×. Also, the spatial resolution for the plutonium dioxide particle was 302.7 ± 4.6 µmFWHM due to the distance between the plutonium dioxide particle and the ZnS(Ag) scintillator. The influence of beta particles was negligible, and alpha particles were discernible in the alpha–beta particle contamination. The equivalent volume diameter of the plutonium dioxide particle was calculated from the measured count rate. These results indicate that the developed alpha imager is effective in the plutonium dioxide particle measurements at the working sites of nuclear facilities for internal exposure dose evaluation.

## Introduction

Plutonium isotopes, such as ^238^Pu, ^239^Pu, and ^240^Pu (alpha emitters), are used as a mixed oxide (MOX) fuel in thermal reactors and fast reactors^1^. Alpha contamination is present in part of the field of decommissioning of nuclear power stations^2^, such as the Fukushima Daiichi Nuclear Power Station (FDNPS). The plutonium isotopes form oxide particles (PuO_2_ particles, also called hot particles), with a diameter of a few micrometers^3^. Considering internal exposure when a worker inhales the PuO_2_ particle at the site of a nuclear facility is crucial. The internal exposure dose calculation of the worker depends on the particle diameter distribution defined as activity median aerodynamic diameter (AMAD)^4^. These particles are deposited in the human respiratory tract, depending on their size. The default value that the International Commission on Radiological Protection (ICRP) recommends for the AMAD is 5 μm^5^. Therefore, it is necessary to determine the value of AMAD of the PuO_2_ particle from the viewpoint of radiation protection.

Conventionally, solid-state nuclear track detectors, such as CR-39 detectors, have been used to evaluate the diameters of PuO_2_ particles by counting the number of tracks^6,7^. The usage technique of CR-39 detectors is being improved continuously. These detectors require an etching process to identify the number of tracks after the exposure to alpha particles. During the etching process, the CR-39 detectors are inserted for hours in a NaOH solution. Next, the number of tracks is measured using an optical microscope. As this process takes time and requires a specific tank and equipment for the use of the NaOH solution, CR-39 detectors are not suitable for real-time utilization at a working site.

Autoradiographs of plutonium particles can be traced using an imaging plate (IP)^8,9^. However, as it requires setting in a reader after the exposure, real-time imaging is also not possible. Moreover, the IP signal intensity gradually decreases after exposure (fading effect)^10^, making it difficult to quantify the radioactivity of plutonium particles without correction of the fading effect. Additionally, the spatial resolution is worse than a CCD-camera based alpha imager for PuO_2_ particle imaging^11^. On one hand, because the IP is sensitive to alpha, beta, and gamma rays, it is difficult to distinguish alpha particles from the background beta particles and gamma rays.

Alpha particle imaging detectors using position sensitive photomultiplier tubes (PSPMTs) and silicon photomultipliers (SiPMs) have also been developed in the past^12–15^. They can obtain alpha particle counts in real-time and are used to estimate PuO_2_ particle diameters. However, it is desirable to acquire optical images and alpha particle imaging simultaneously. The optical images are useful information for the subsequent analysis using different methods if the exact location of the PuO_2_ particles is to be determined.

On the other hand, high-resolution alpha imagers have been considered for clinical applications, such as the targeted alpha therapy (TAT), mainly described with ^211^At being injected into a patient. Some high-resolution alpha imagers have recently been developed for the TAT. Bäck et al. have developed an alpha imager that combines a high-sensitivity charged-coupled device (CCD) camera with a ZnS(Ag) sheet. This alpha imager can be applied to identify the alpha particle distribution of ^211^At in the organs of mice^16^. The spatial resolution of this imager was 35 ± 11 μm. Miller et al. developed an alpha camera named iQID camera, and the spatial resolution of ∼ 20 µm FWHM was achieved^17^.

Yamamoto et al. have developed a high-resolution alpha imager using a CCD camera and an optical fiber structure scintillator plate, which can visualize alpha particle trajectories with an ultrahigh spatial resolution (~ 11 µm)^18^.

Pratx et al. developed radioluminescence microscopy using a CdWO_4_ scintillator plate and a microscope to capture the individual beta particle track^19^. Radioluminescence microscopy is a new technique for visualizing radionuclides in live cells and acquires a sequence of frames with short exposure to count the ionization tracks.

Kurosawa et al. have developed a high-resolution alpha imager by combining a complementary metal–oxide–semiconductor (CMOS) camera with a microscope^20^. This alpha imager can choose its zoom range and is capable of an image resolution of 2.1 µm for optical imaging. As the spatial resolution for alpha particles is better than 100 µm, which is a better resolution than those of the conventional alpha imagers used for AMAD value measurements at the workplaces of nuclear facilities, such as the IP detectors, an alpha imager using a camera and a microscope can be used to determine in real time the diameters of PuO_2_ particles.

Alpha and beta contamination existed in the FDNPS site. For use at the FDNPS site, the alpha imager must be sensitive to alpha particles only. In this study, we develop a high-resolution alpha imager using a CCD camera and an optical microscope based on previous studies and applies the technique to measure an actual PuO_2_ particle.

## Results

### Optical images

**Figure 1:**
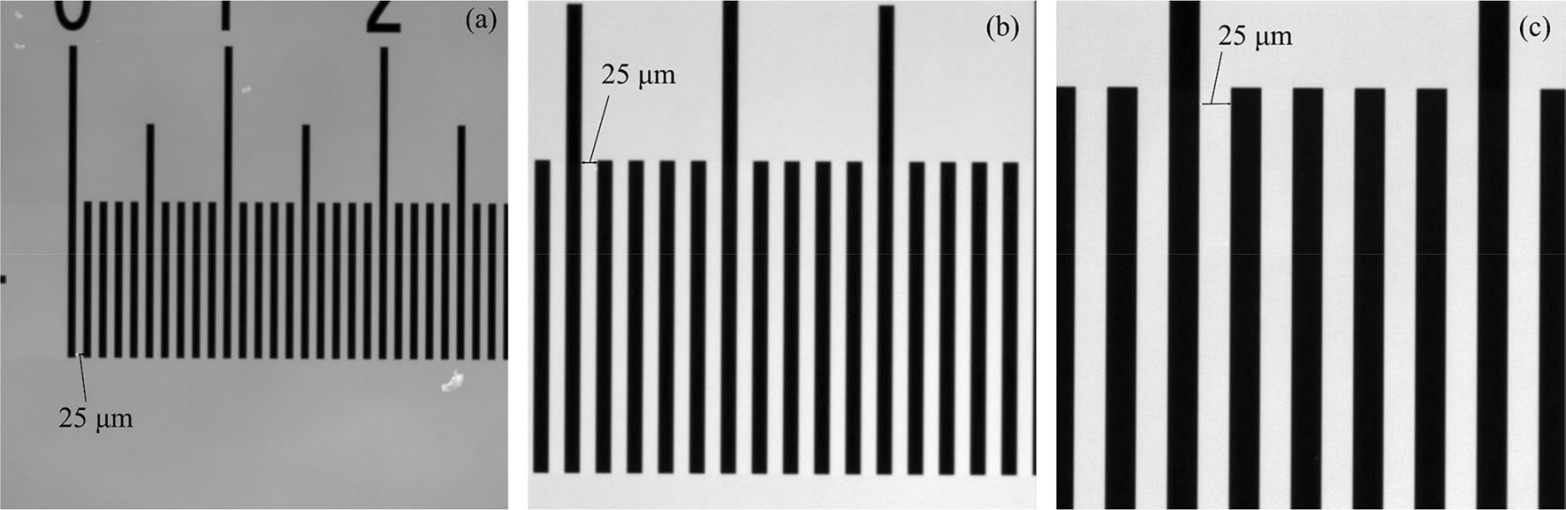
Optical images of the calibration sample at zoom ranges of (**a**) 5 ×, (**b**) 10× , and (**c**) 20 ×. The slit width is 25 µm.

Figure 1 presents the optical images of the calibration sample with the zoom ranges 5 ×, 10 ×, and 20 ×. The slit width of the calibration sample was 25 µm. The relation between the image resolution and FOV with the same zoom ranges is presented in Table 1. A trade-off could be observed between the image resolution and FOV. For instance, at the zoom range of 5 ×, the resolution and FOV were 3.28 µm/pixel and 1679.4 µm × 1679.4 µm, respectively, whereas when 20 × was used, the corresponding values were 0.81 µm/pixel and 412.9 µm × 412.9 µm.

**Table 1:** The relation between the image resolution and FOV with the zoom ranges of 5 ×, 10 ×, and 20 ×.

Zoom	Resolution (distance/pixel)	Field of view
5 ×	3.28 µm	1679.4 µm × 1679.4 µm
10 ×	1.63 µm	835.0 µm × 835.0 µm
20 ×	0.81 µm	412.9 µm × 412.9 µm

### Radiation imaging

#### Alpha source imaging

**Figure 2:**
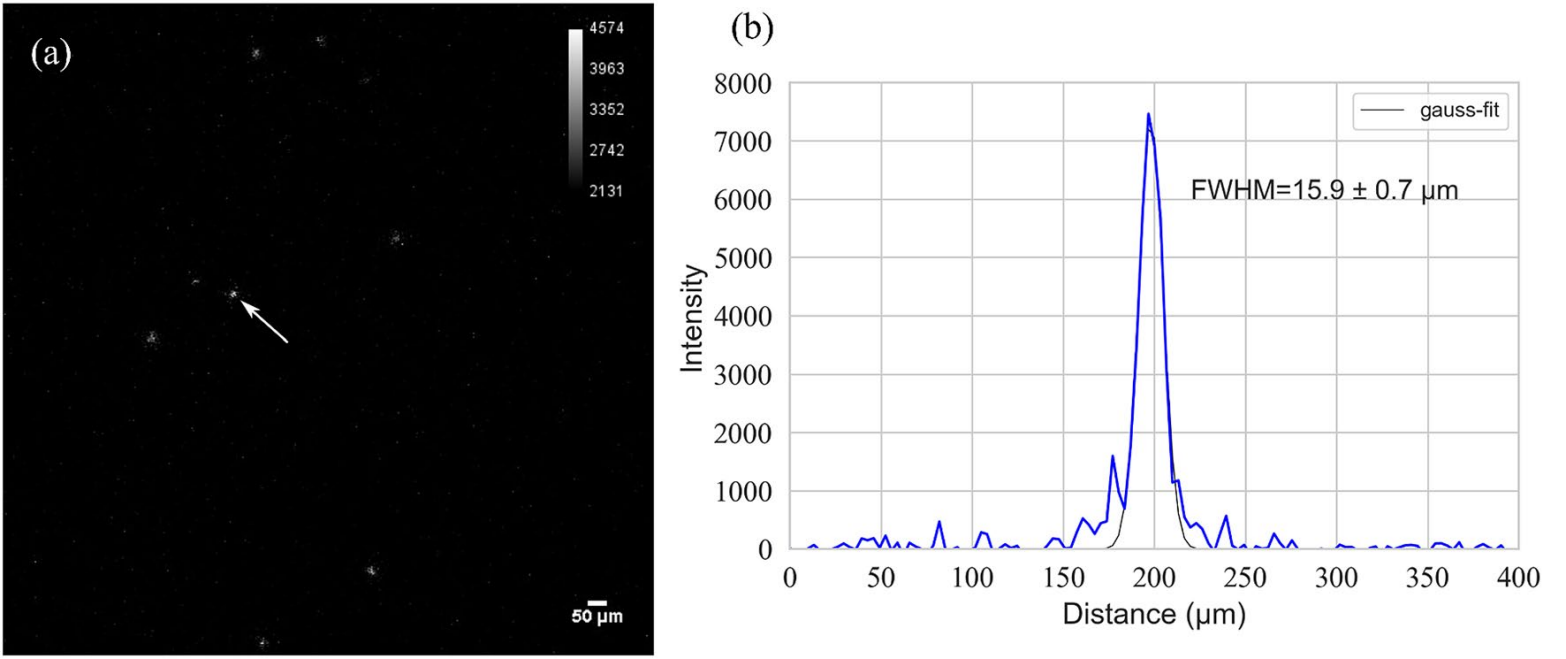
(**a**) Image of an alpha particle captured using the high-resolution alpha imager with a zoom range of 5 ×. (**b**) Intensity profile of the alpha particle with a zoom range of 5 × .

Figure 2 presents the image of an alpha particle measured using the high-resolution alpha imager with a zoom range of 5 × and along with the particle’s intensity profile. The full width at half maximum (FWHM) of the alpha particle intensity profile shown in Fig. 2(b) was evaluated using the Gaussian fitting, at 15.9 ± 0.7 μm with a zoom range of 5 × . The average spatial resolution of the multiple alpha particles was evaluated to be 16.2 ± 2.2 μm with a zoom range of 5 ×.

**Figure 3:**
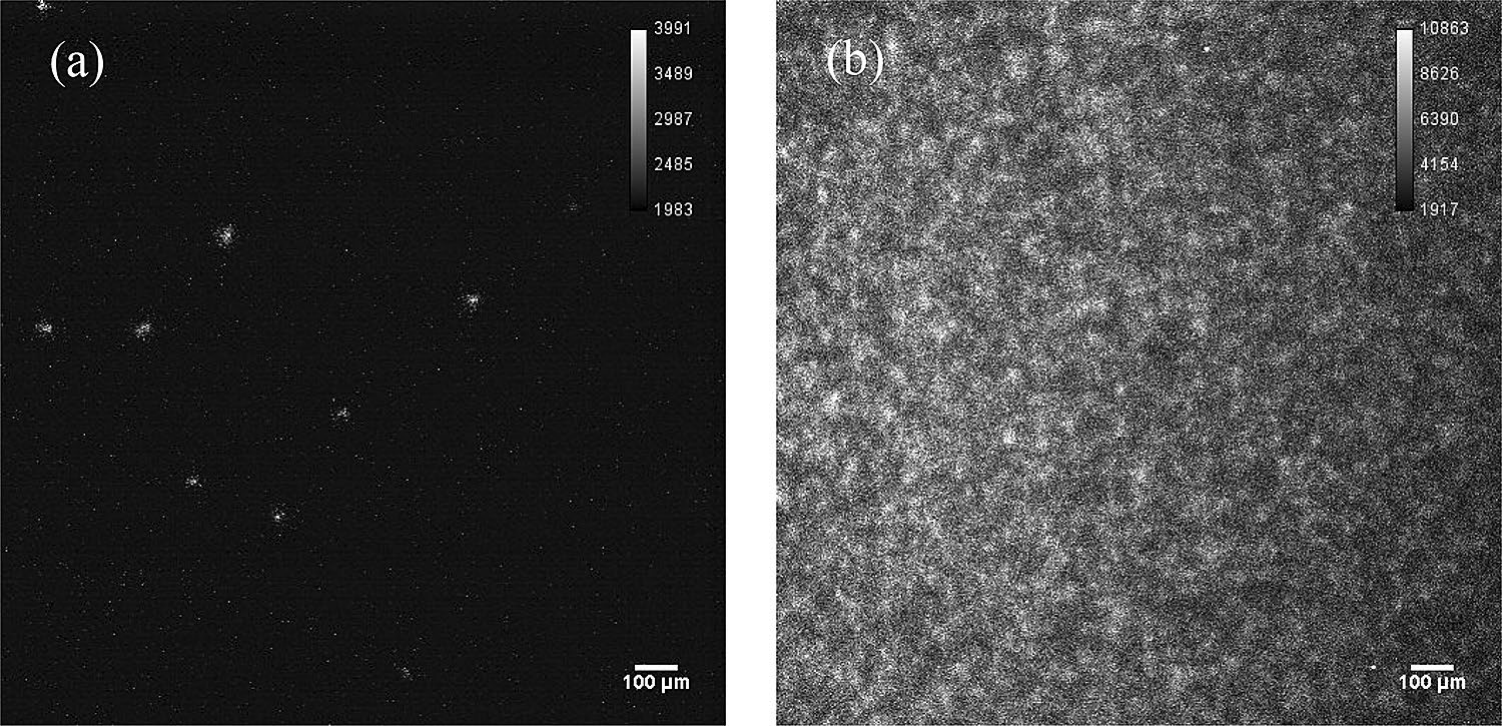
Image of an alpha particle from the active area of the ^241^Am alpha source obtained with the zoom range of 5 × using (a) the 50-ms sequence mode and (b) the 10-min acquire mode. The images of using the “Sequence mode” with 50-ms intervals can be found as Supplementary Fig. S1 online.

Figure 3a presents the image of an alpha particle from the active area of the ^241^Am alpha source obtained using the “Sequence mode” with 50-ms intervals. The entire area of the image was the active area of the ^241^Am alpha source. The alpha particles were confirmed at different locations within 50 ms. Similarly, Fig. 3b presents the image of alpha particles obtained using the “Acquire mode” for 10 min. Here the alpha particles were distributed all over the image.

#### Beta source imaging

**Figure 4:**
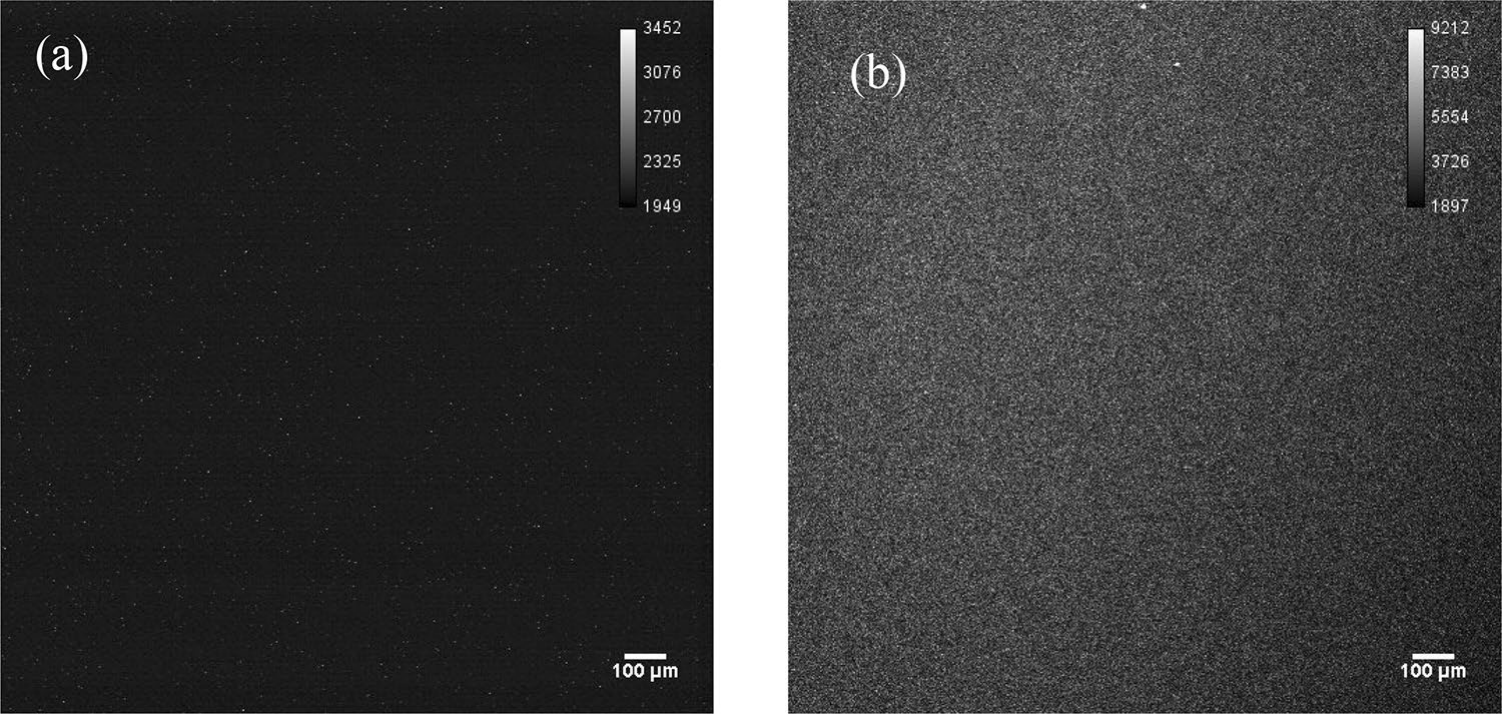
Image of an alpha particle from the active area of the ^90^Sr/^90^Y beta source obtained with the zoom range of 5 × using (**a**) the 50-ms sequence mode and (**b)** the 10-min acquire mode. No spot is observed. The images of using the “Sequence mode” with 50-ms intervals can be found as Supplementary Fig. S2 online.

Figure 4a presents the image from the active area of the ^90^Sr/^90^Y beta source obtained using the sequence mode with 50-ms intervals. The entire area of the image was the active area of the ^90^Sr/^90^Y beta source. No particle was confirmed within 50 ms. Figure 4b presents the image obtained using the acquire mode for 10 min. Similarly, no particle could be observed on the image. These results indicate that the influence of beta particles is negligible and that alpha particles can be distinguished against them.

#### *PuO*_*2*_* particle*

**Figure 5:**
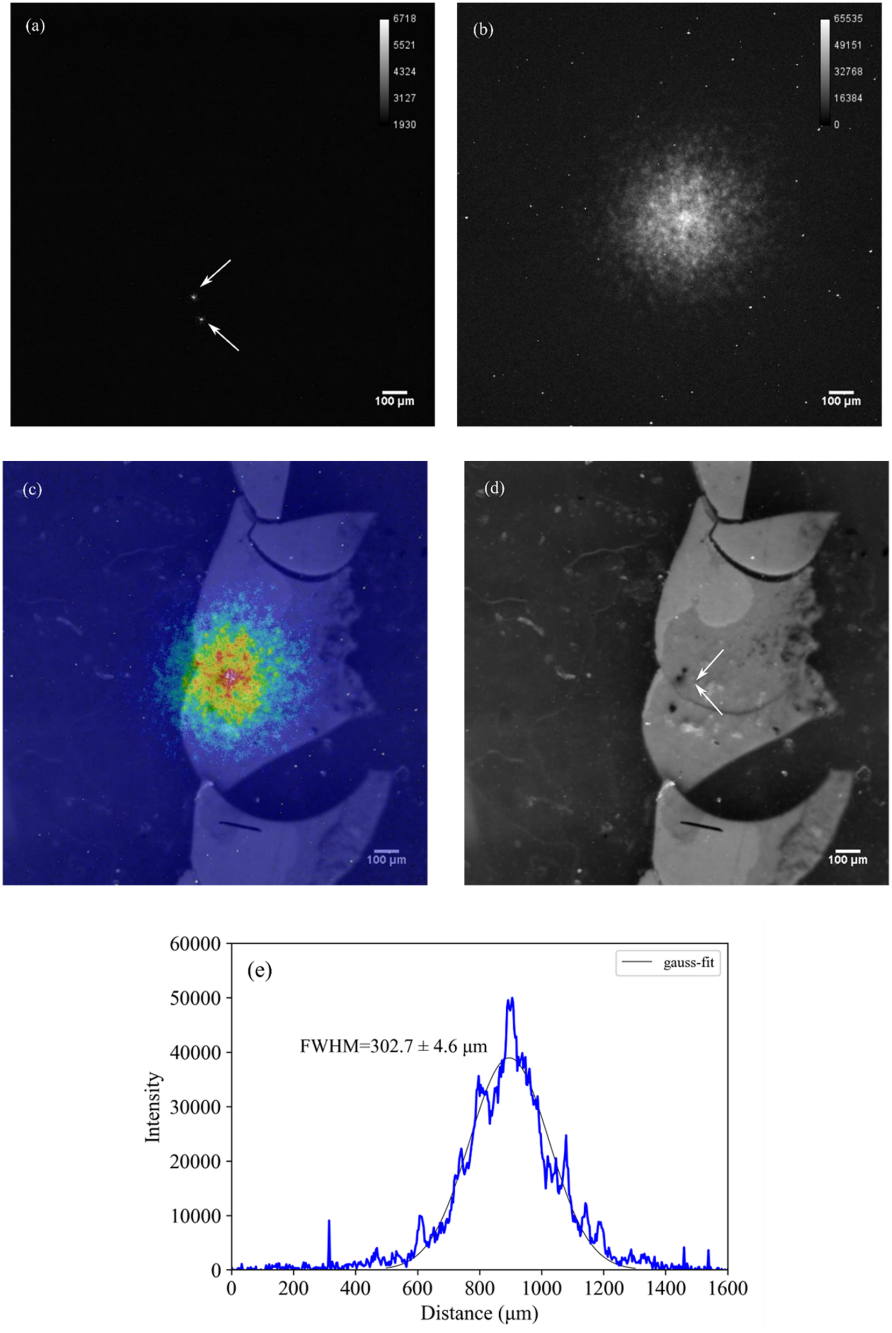
Alpha particles emitted from a plutonium particle with a zoom range of 5 × using (**a**) the 50-ms, (**b**) 30-min acquisitions, (**c**) superimposition of the optical image and alpha particle image, (**d**) the optical image, and (**e**) intensity profile of the alpha particle emitted from a plutonium particle. The arrows in (**a**) show the individual alpha particle locations. The arrows in (**d**) show the PuO_2_ particle location. The images of using the “Sequence mode” with 50-ms intervals can be found as Supplementary Fig. S3 online.

Figure 5a presents the alpha particles emitted from the PuO_2_ particle with the 50-ms intervals of the sequence mode. The locations of the individual alpha spots can be identified in real time. Figure 5b presents the alpha particles emitted from the PuO_2_ particle using the acquire mode for 30 min. The number of alpha particles corresponded with that measured using the commercial ZnS(Ag) scintillation counter, at a detection efficiency (*D*_*eff*_) of 99.3% based on Eq. (1). Figure 5c shows a superimposition of the optical image and alpha particle image, and Fig. 5d shows the optical image with an arrow showing the position of the PuO_2_ particle. Figure 5e shows an intensity profile of the alpha particle emitted from a plutonium particle. The spatial resolution for the PuO_2_ was 302.7 ± 4.6 µmFWHM. The spatial spreading of the alpha particles in Fig. 5e depends on the distance of the thin polyethylene film between a plutonium particle and the ZnS(Ag) scintillator.

#### Radon-222 progeny

**Figure 6:**
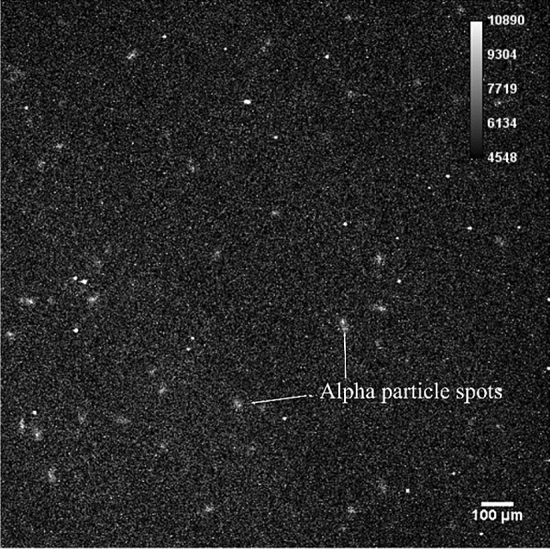
Alpha particles emitted from the ^222^Rn progeny with a zoom range of 5 × using the 30-min acquisition.

Figure 6 shows the alpha particles emitted from the ^222^Rn progeny using the acquired mode for 30 min. Alpha particles are distributed throughout the image. The distribution of alpha particles of ^222^Rn was different from the PuO_2_ particles, which correlated well with the results obtained from other alpha imaging detectors^21^.

### Evaluation of the equivalent volume diameter (*d*_e_) of a PuO_2_ particle

**Figure 7:**
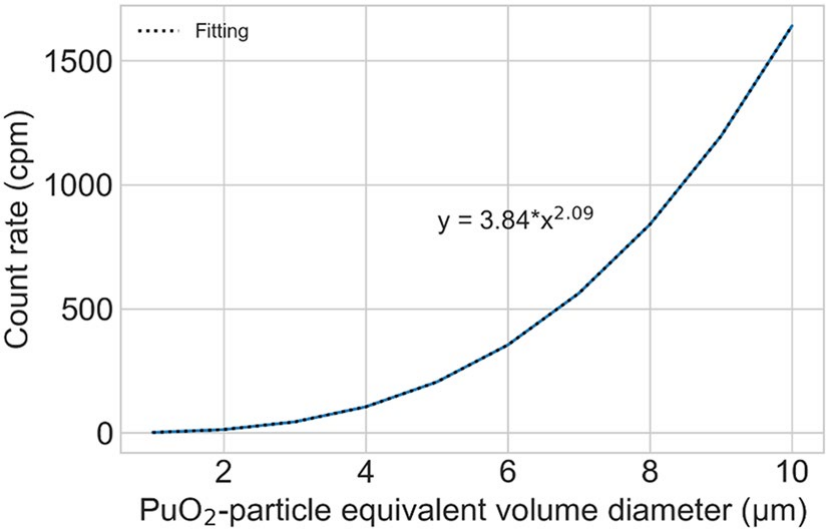
*CF* curve for the conversion of the measured alpha counts into *d*_*e*_ calculated using the Monte Carlo simulation.

Figure 7 presents the conversion factor (*CF*) curve used for evaluating the diameter of a PuO_2_ particle as a function of the count rate calculated using the Monte Carlo simulation. The equation of the curve can be approximated as y = 3.84 × (*d*_*e*_^2.09^). Where the alpha counts detected in the experiment were 214.3 ± 5.2 cpm, the *d*_*e*_ of the PuO_2_ particle was evaluated to be 6.9 μm. Accordingly, the equivalent aerodynamic diameter (*d*_*ae*_) was determined to be 17.4 μm using Eq. (6).

## Discussion

We have developed the high-resolution alpha imager, measured the actual PuO_2_ particle, and calculated its *d*_*e*_ and *d*_*ae*_. At a zoom range of 5 ×, the resolution was 3.28 µm/pixel, but with a larger FOV of 1679.4 × 1679.4 µm. Thus, lower zoom ranges like 5 × appropriate the identification of the locations of PuO_2_ particles on a sample.

Using the sequence mode allowed the measurement of individual alpha particle spots with a high spatial resolution (~ 17 µm FWHM). This feature is useful in evaluating the activity and diameter of PuO_2_ particles. Moreover, the electron-multiplying charged-coupled device (EMCCD) camera is capable of changing the exposure time intervals from 13.9 ms to 2 h. The short time interval measurement helps avoid the overlapping of multiple alpha particles, as well as accurately measures the number of alpha particle spots. Conventional detectors, such as the CR-39 and IP detectors, are passive detectors that need long exposure times; if the exposure time is not appropriate, then multiple alpha particles will overlap during the measurement. The “no-fading effect,” as in the case of IP detectors, was another advantage of our developed alpha imager.

Further, the proposed alpha imager demonstrates no beta sensitivity, even with the measurement of the 1-MBq ^90^Sr–^90^Y beta source. As the ZnS(Ag) scintillator was only 3.25-mg/cm^2^ (approximately 8-μm) thick, there were a few possibilities that beta particles absorb their energy in the scintillator. For example, the range of a 5.5-meV alpha particle is approximately 10 mg/cm^2^ in the ZnS(Ag) scintillator. In the FDNPS site, where beta contamination activities were much higher than those of alpha, our alpha imager could prove useful in measuring the alpha-emitting particles.

Moreover, the measured *d*_*e*_ and *d*_*ae*_ values of the PuO_2_ particle were 6.9 and 17.4 μm, respectively, the latter being in the 1.0–28.0-μm range of the previous IP detector measurements^22^. The calculated CF values for PuO_2_ particles were calculated in the range *d*_*e*_ of 1–10 μm (*d*_*ae*_ of 2.7–25.4 μm). The AMAD value of 5 μm is representative of workplace aerosol value^23^. The value of 5 μm is within this range.

In a facility like the one from which we obtained our samples, the PuO_2_ particles generated by the dismantling of process equipment were measured using a cascade impactor^24^. The results were distributed in the range of 1–11 μm measured using a cascade impactor. Our measured results (*d*_*e*_, equivalent particle size = 6.9 μm) are well within that range.

The abovementioned methods are useful in determining the AMAD values in real time at the working site of nuclear facilities, which will help evaluate the internal exposure dose of workers instantaneously in case of internal exposure accidents.

## Conclusions

The proposed high-resolution alpha imager is a combination of a ZnS(Ag) scintillator sheet, an optical microscope, and an EMCCD camera. The alpha imager is capable of visualizing individual alpha particle spots in real time with a high spatial resolution (~ 16.2 ± 2.2 μm with a zoom range of 5 ×). As the influence of beta particles was found to be negligible, alpha-emitting particles can be distinguished in a mixed alpha and beta contamination environment, as in any FDNPS sites. The values of the *d*_*e*_ and *d*_*ae*_ of the PuO_2_ particle were calculated from the measured count rate. Based on these results, the developed alpha imager will be useful for PuO_2_ particle measurements at the working sites of nuclear facilities for instantaneous internal exposure dose evaluation.

## Methods

### High-resolution alpha imager

**Figure 8:**
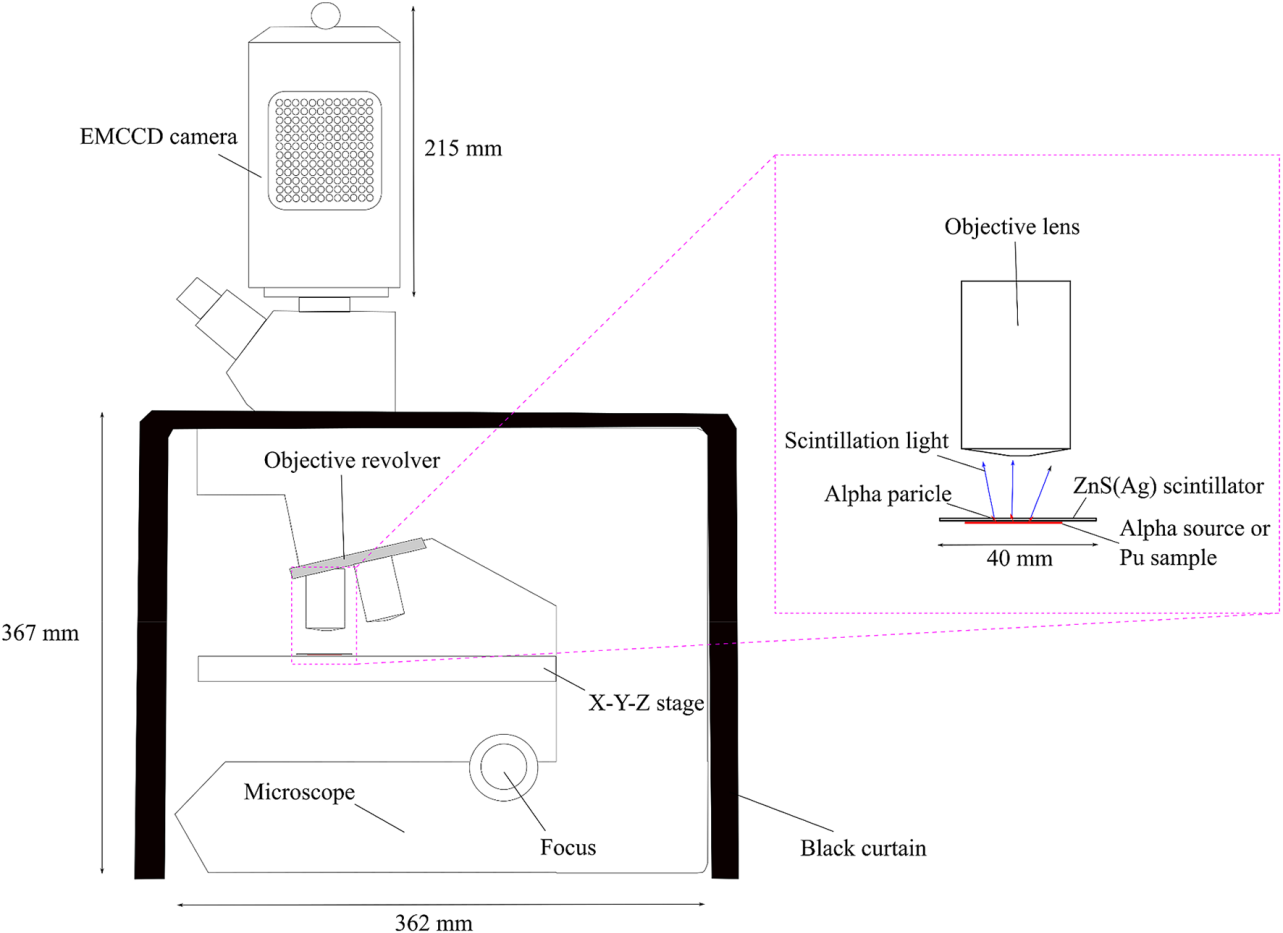
Schematic drawing of the developed high-resolution alpha imager.

**Figure 9:**
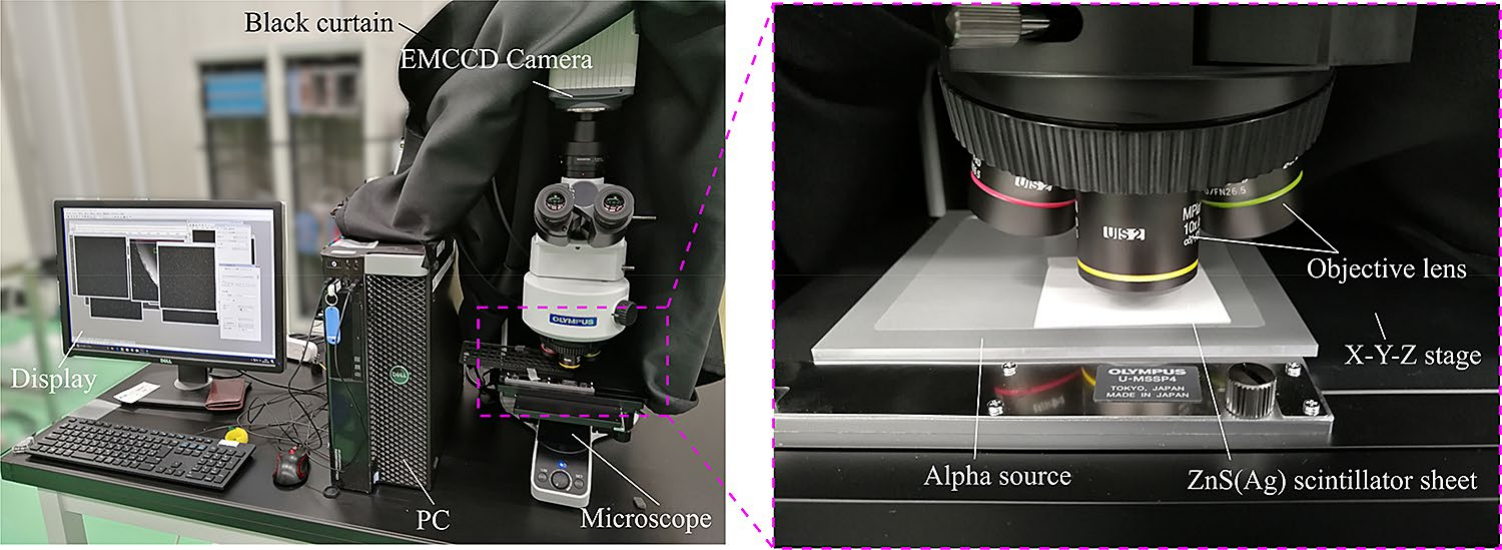
The developed alpha imager.

Figure 8 presents a schematic drawing of the high-resolution alpha imager developed using a ZnS(Ag) scintillator sheet (EJ-440, Eljen Technology, TX, USA), an optical microscope (BX53MRF-S, Olympus Corp., Tokyo, Japan), and an EMCCD camera (ImagEM X2, Hamamatsu Photonics k.k., Shizuoka, Japan) (Fig. 9). The thickness of the ZnS(Ag) scintillator was 3.25 mg/cm^2^ (approximately 8 μm). The EMCCD camera was mounted on the optical microscope to capture the images of scintillation light or optical light. Output signals from the EMCCD camera were transferred to a PC using an IEEE1394 cable. A data acquisition software with “Acquire mode,” to identify a time-integrated image, and “Sequence mode,” to identify a series of single images, was used. The CCD camera was automatically cooled at − 65 °C after it was switched on to reduce thermal noise. The optical microscope was covered with a black curtain to block unwanted light from the outside.

### Optical imaging

The optical images of the calibration sample were captured within the zoom range of 5 × –20 × using the alpha imager to evaluate its image resolution. The optical image was taken under room light conditions.

### Radiation imaging

#### Alpha source imaging

**Figure 10:**
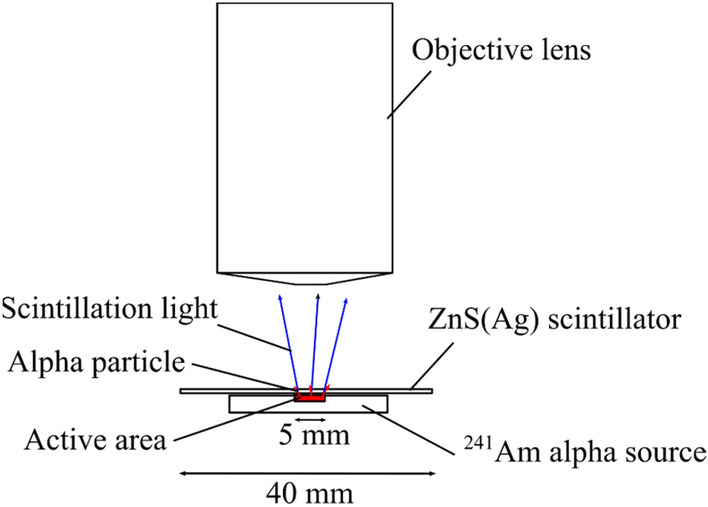
Schematic drawing of the alpha source imaging.

We used the alpha imager to measure 5.5 meV alpha particles emitted from the ^241^Am alpha source. A schematic drawing of the alpha source imaging is presented in Fig. 10. The source has a 5-mm-diameter active area and a 3.37-kBq activity. Alpha particles deposited their energies in the scintillator, and the light emitted from the scintillator that passed through the lens was captured by the CCD camera.

#### Beta source imaging

**Figure 11:**
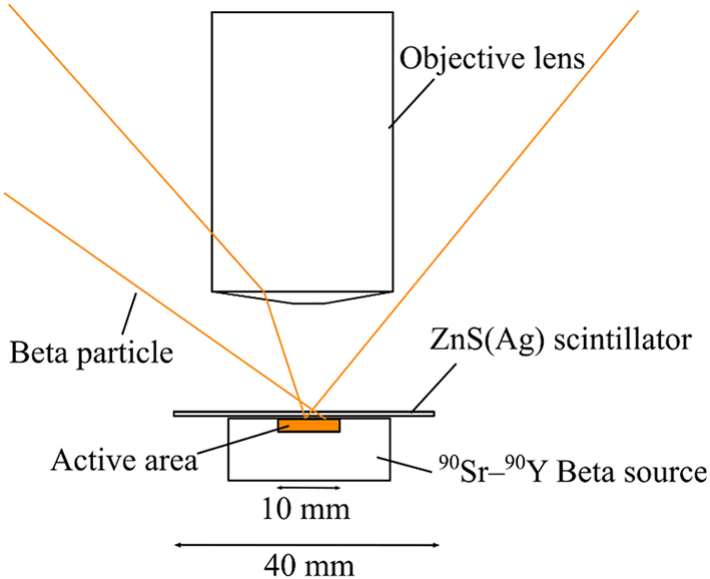
Schematic drawing of the beta source imaging.

Because alpha contamination is sometimes accompanied by beta contamination and needs to be distinguished, it is important to evaluate the beta sensitivity to the high-resolution alpha imager. Figure 11 presents the schematic drawing of the beta source imaging. Using the alpha imager, we measured a 1-MBq ^90^Sr − ^90^Y beta source with a 10-mm-diameter active area. Also, the ZnS(Ag) scintillator was placed onto the beta source.

#### *PuO*_*2*_* particle imaging*

**Figure 12:**
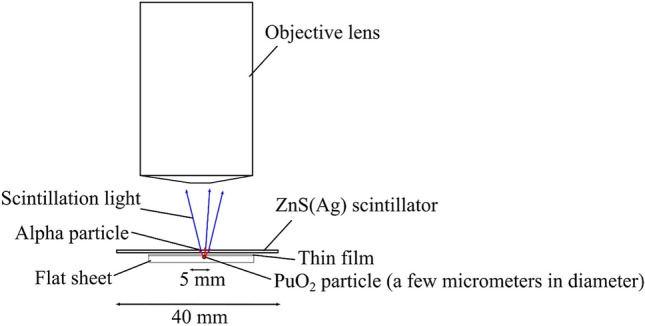
Schematic drawing of the PuO_2_ particle imaging.

Using the same alpha imager, we measured a PuO_2_ particle obtained from a MOX fuel facility. Figure 12 presents a schematic drawing of the PuO_2_ particle imaging. A single PuO_2_ particle was attached on a flat sheet and covered with a thin-film sheet. The PuO_2_ particle was then covered with a thin polyethylene film. The alpha source and the plutonium particle were set on the X–Y–Z stage. Subsequently, the ZnS(Ag) scintillator sheet was placed on top of the thin polyethylene film. Moreover, we measured the same PuO_2_ particle using a commercial ZnS(Ag) scintillation counter (JDC-817, Hitachi Aloka Medical, Tokyo, Japan), having a 100% detection efficiency for alpha particles with a 2 $\pi$ angle. The detection efficiency of the alpha imager was evaluated by comparing with the measured count rate using the commercial ZnS(Ag) scintillation counter according to the following equation:

1 $${D}_{eff}=\frac{{C}_{alpha\, imager}}{{C}_{ZnS\left(Ag\right)\,counter}}\times 100$$

where *C*_*alpha imager*_ and C_*ZnS(Ag) counter*_ represent the measured count rates of the alpha imager and the commercial ZnS(Ag) scintillation counter, respectively.

#### Radon-222 imaging

**Figure 13:**
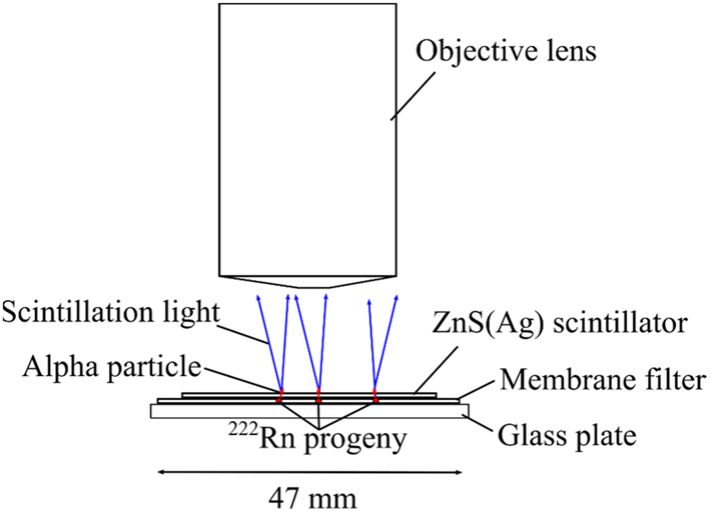
Schematic drawing of the ^222^Rn progeny imaging.

^222^Rn progeny was also measured using the alpha imager. Figure 13 shows a schematic drawing of ^222^Rn progeny imaging. The radon progeny were collected using an air sampler (DSM-361, Hitachi Aloka Medical, Ltd. Mitaka, Tokyo, Japan) through a 47-mm diameter membrane filter (Toyo Roshi Kaisha, Ltd. Tokyo, Japan). Air sampling was performed in an environment with a ^222^Rn concentration of 200 Bq/m^3^ for ~ 4 h, and filter paper was collected after sampling. The membrane filter was fixed on a glass plate with a diameter of 50 mm × 50 mm, and the ZnS(Ag) scintillator was placed on top of the membrane filter.

### Method for auto-identification of alpha particle spots

**Figure 14:**
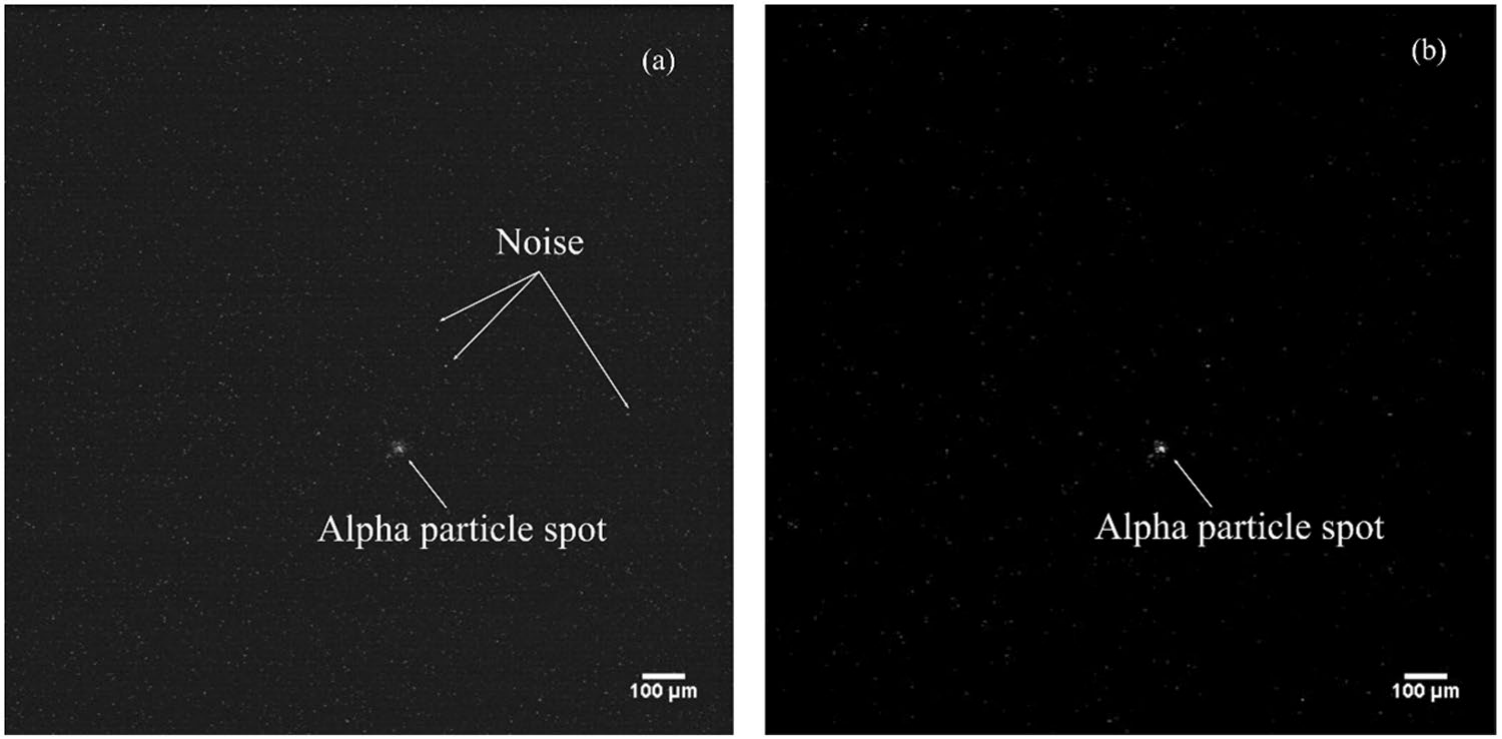
Example of an alpha particle spot: (**a**) before and (**b**) after the image processing. Noise signals are observed randomly distributed in the image.

As presented in Fig. 14, the alpha particle was confirmed as a spot when measuring with the alpha imager. The alpha particle spots were auto-identified by processing to acquire images as follows.

First, we applied a 2D Gaussian filter (smoothing filter) to remove the noise distributed in the image caused by the image sensors. We used the OpenCV library to convolve with an image the 5 × 5 Gaussian filter kernel:

2 $$K=\frac{1}{25}\times \left[\begin{array}{ccccc}1& 4& 6& 4& 1\\ 4& 16& 24& 16& 4\\ 6& 24& 36& 24& 6\\ 4& 16& 24& 16& 4\\ 1& 4& 6& 4& 1\end{array}\right]$$

After applying the filter to the image, we employed a binary processing onto the image to detect the edge of the alpha particle spot. Finally, we obtained the number of contours (alpha particles).

### Evaluation of d_***e***_ and ***d***_***ae***_ of the PuO_***2***_ particle

The value of the *d*_*e*_ and *d*_*ae*_ of a PuO_2_ particle is crucial for evaluating the AMAD value in the internal exposure dose calculation. *d*_*e*_ is defined as the diameter of the spherical particle with the same volume as the particle considered^25^.

**Figure 15:**
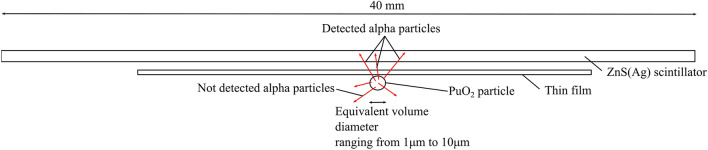
Geometry of the Monte Carlo simulation setup, which is the same as that of the measurement. The equivalent volume of the PuO_2_ particle is varied from 1 to 10 µm.

First, *d*_*e*_ was calculated from the number of detected counts of the alpha particles measured by the alpha imager. We obtained *d*_*e*_ by calculating a *CF* to convert the number of detected counts by our imager into the *d*_*e*_ of a PuO_2_ particle using a Monte Carlo particle and heavy ion transport code system (PHITS) ver. 2.8 simulation^26^. *CF* was used to convert the measured count rate to the equivalent volume diameter of PuO_2_ particles. We reproduced a setup in a Monte Carlo simulation code which is similar to the actual PuO_2_ particle imaging. Figure 15 presents the geometry of the simulation. The alpha particles were emitted to isotropic angles from the PuO_2_ particle. The Pu isotopic composition of the PuO_2_ particle was based on a MOX fuel that we measured using our imager. The alpha activities *A*_*i*_ of the Pu isotope were determined by multiplying the specific activity *SA*_*i*_ of each isotope by the isotopic composition *IC*_*i*_ as follows:

3 $${A}_{i}={SA}_{i}\times {IC}_{i}.$$

Accordingly, the diameters of the Pu particle varied from 1.0 to 10.0 μm in the simulation. The density of the PuO_2_ particle was 11.46 g/cm^3^. *CF* was calculated using

4 $$CF=\frac{{d}_{e}}{{N}_{alpha}},$$

where *N*_*alpha*_ denotes the number of detected alpha counts measured using our imager.

Particles suspended in the atmosphere move differently at different densities. Therefore, to compare the properties of particles of different densities, the *d*_*ae*_ value should be calculated. *d*_*ae*_ can be numerically solved from *d*_*e*_ using the equation^22^,

5 $${d}_{ae}={d}_{e}\times \sqrt{\frac{\rho C({d}_{e})}{\chi {\rho }_{0}C({d}_{ae})}}$$

6 $$C\left(d\right)=1+\left(\frac{\lambda }{d}\right)\left\{2.54+0.800exp\lceil-0.55(\frac{d}{\lambda })\rceil\right\},$$

where λ = 0.0712 μm; χ and ρ are the particle shape factor (1.8, cited from the experimental data^27^) and mass density, respectively; ρ_0_ is the unit density (1 g/cm^3^); and *C(d)* is the slip correction (often called the Cunningham factor)^22^. The value of *d*_*ae*_ is defined by ICRP as the value at which 50% of the airborne activity in a specified aerosol is associated with particles greater than the AMAD^28^.

## Supplementary Information

Supplementary Information 1.

Supplementary Information 2.

Supplementary Information 3.

Supplementary Information 4.
