# Supplementary material for: Plutonium dioxide particle imaging using a high-resolution alpha imager for radiation protection
Source: Sci Rep. 2021 Mar 15;11:5948. doi: 10.1038/s41598-021-84515-z (PMC7961019; doi:10.1038/s41598-021-84515-z)
Supplement: Supplementary file 1 — Supplementary Information 1. [file 41598_2021_84515_MOESM1_ESM.docx]

**Plutonium dioxide particle imaging using a high-resolution alpha imager for radiation protection**

Yuki Morishita^1,*^, Shunsuke Kurosawa^2,3^, Akihiro Yamaji^2,3^, Masateru Hayashi^4^, Makoto Sasano^4^, Taisuke Makita^4^, and Tetsushi Azuma^4^

Supplementary Information


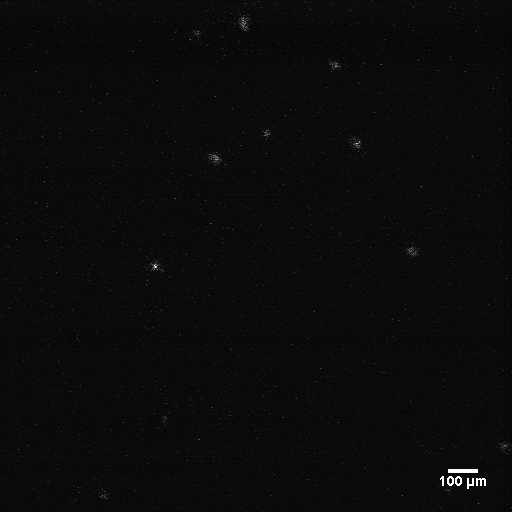


Figure 3 Supplementary Figure S1


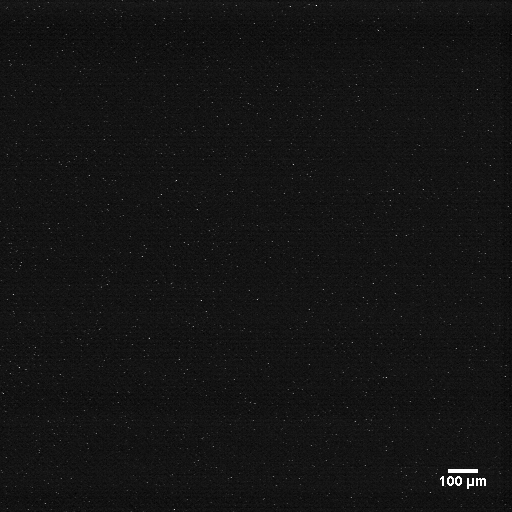


Figure 4 Supplementary Figure S2


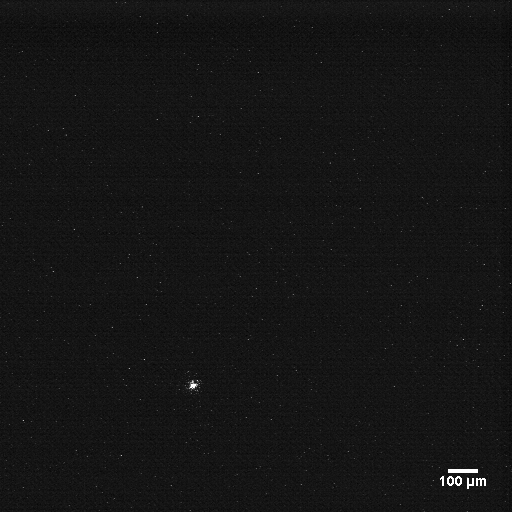


Figure 5 Supplementary Figure S3
