# Supplementary figures and images for: Plutonium dioxide particle imaging using a high-resolution alpha imager for radiation protection
Source: Sci Rep. 2021 Mar 15;11:5948. doi: 10.1038/s41598-021-84515-z (PMC7961019; doi:10.1038/s41598-021-84515-z)

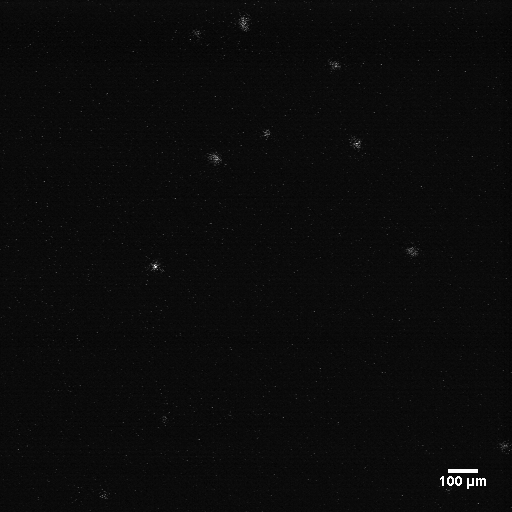

Supplement: Supplementary file 2 — Supplementary Information 2. [file 41598_2021_84515_MOESM2_ESM.gif]

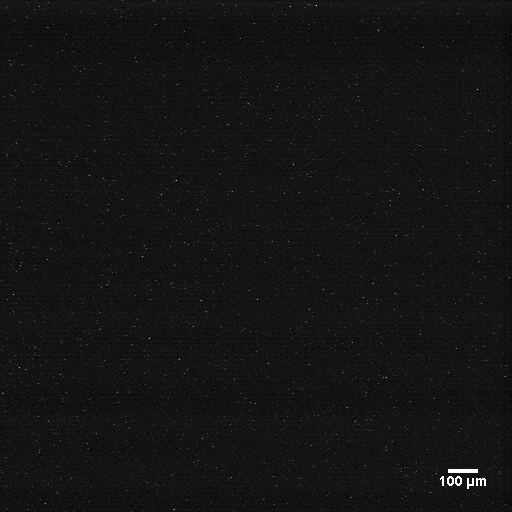

Supplement: Supplementary file 3 — Supplementary Information 3. [file 41598_2021_84515_MOESM3_ESM.gif]

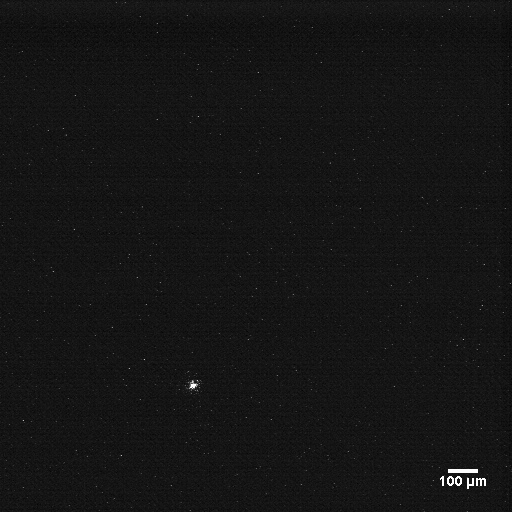

Supplement: Supplementary file 4 — Supplementary Information 4. [file 41598_2021_84515_MOESM4_ESM.gif]
